# Combined Toxicity of Nitro-Substituted Benzenes and Zinc to Photobacterium Phosphoreum: Evaluation and QSAR Analysis

**DOI:** 10.3390/ijerph16061041

**Published:** 2019-03-22

**Authors:** Shengnan Zhang, Limin Su, Xujia Zhang, Chao Li, Weichao Qin, Dongmei Zhang, Xiaoxia Liang, Yuanhui Zhao

**Affiliations:** 1 School of Environment, and State Environmental Protection Key Laboratory of Wetland Ecology and Vegetation Restoration, Northeast Normal University, Changchun 130117, China; zhangsn432@nenu.edu.cn (S.Z.); zhangxj957@nenu.edu.cn (X.Z.); lic932@nenu.edu.cn (C.L.); qinwc425@nenu.edu.cn (W.Q.); zhangdm941@nenu.edu.cn (D.Z.); liangxx512@nenu.edu.cn (X.L.); 2 College of Geographical Sciences, Harbin Normal University, Harbin 150025, China

**Keywords:** joint toxicity, QSAR, substituted benzenes, *Photobacterium phosphoreum*, zinc

## Abstract

The single toxicity (IC_50_) of zinc (Zn) and 11 nitro-substituted benzenes to *Photobacterium phosphoreum* were determined, respectively. On basis of single toxicity, the joint toxicity of binary mixtures of Zn and 11 nitro-substituted benzenes at different Zn concentrations of 0.2 IC_50_, 0.5 IC_50_, and 0.8 IC_50_ were measured. The joint toxicity was evaluated by toxic unit (TU) and additive index (AI) methods. The results indicated that the joint toxicity was not only depending on the Zn concentrations but also on the substituted groups of nitro-substituted benzenes. The quantitative structure-activity relation (QSAR) equations were developed and the results showed that the toxicity of nitro-substituted benzenes has different joint effect at the different Zn concentrations. At the Zn concentration of 0.2 IC_50_, the binary joint effects were mainly antagonism and the joint toxicity was negatively related to descriptors called VE2_B(p) and TIC3. At the Zn concentration of 0.5 IC_50_ and 0.8 IC_50_, the binary joint effects were mainly antagonism and simple addition, and the joint toxicity was related to the same descriptor Eig06_ AEA(dm). It indicated that the joint toxic actions were similar when combined at the medium and high concentrations of Zn.

## 1. Introduction

It is obviously that the risks of chemical pollutants are mostly evaluated on the single toxicity value of a chemical and the adverse effects or single actions of chemicals have received extensive attention [1,2,3,4]. Although it is crucial to identify and characterize harmful effects from pollutants on different organisms, it is incontestable that those results are not enough to tell what happens in the real environment [5]. After all, contaminants are introduced into the aquatic ecosystems as complex blends. When the chemicals collectively exist, it is worth taking into account that certain pollutant combinations may interact and result in different interactions toxic effects on ecosystems or organisms [5,6]. Interactions of components in a mixture can cause complex and substantial changes in the apparent properties of its constituents [7]. The joint effect of toxicants could be less than, equal to, or greater than the sum of the effects of the individual toxicants which is often expected, and correspondingly the toxicity of toxicants could be described as antagonistic, additive, or synergistic [8,9]. The use of single toxicity data may fail to predict associated effects and interactions of chemicals in mixture and so is limited to cast light on ecological risk in naturally contaminated aquatic environments [10,11,12,13]. Therefore, realistic safe limits on basis of co-occurrence of chemicals needs to be considered for environmental management.

Nitro-substituted benzenes, used in a broad range of fields as important raw materials and products of chemical industry, have been widely determined in the municipal wastewater and aquatic environments. They have been thought to be an important class of environmental contaminants [14]. Many nitro-substituted benzenes and their transformation products are toxic and some cases even carcinogenic [15,16]. Heavy metals are ubiquitous and persistent pollutants in the environment. They can be easily accumulated in organism by bio-concentration and bio-magnification and finally endanger the health of human beings [17]. Zn, as one of the heavy metals, is extensively used by numerous industries mainly in galvanization and in the manufacture of brass and other alloys, and it is introduced to surface waters due to activities of humans as both point and nonpoint source [18]. Zn is nutritionally essential elements and thus its toxic effects are relatively less in comparison to other more potent nonessential metals. However, it can induce toxic effects on the hematopoietic system, biochemistry, and endocrine system function when exposures exceeded required concentrations [19]. A study found that Zn (II) is toxic for humans at levels of 100–500 mg/d [18]. 

Most studies have been carried out on the combined toxicity between Zn and other heavy metals [20,21,22]. There are also few studies that focus on the joint toxicity of Zn and organic chemicals [23,24]. Liu et al. [23] investigated the joint toxicity of perfluorooctane sulfonate (PFOS) and zinc and the results show that the combined effects of the PFOS and Zn binary mixture to *Limnodrilus hoffmeisteri* were mostly simple addition. Zn and PFOS were also found to evoke some changes in the antioxidant defense system, and a strong self-adaptive ability was noticed after 10d exposure. With the combined treatment of Zn and Cefradine, the bacterial *Pseudomonas fluorescens* YZ2 resistance was improved showing a more significant increase of total superoxide dismutase (SOD) activity and therefore it is found that the binary stress induced the bacterial resistance by regulating SOD activity to eliminate reactive oxidative species (ROS) [24].

Until now, the joint interaction of nitro-substituted benzenes and Zn to organisms in aquatic ecosystem is poorly understood and little knowledge regarding their potential to produce additive toxicity or non-additive toxicity (i.e., antagonism or potentiation) although, as the common contaminants, the mixtures of heavy metal Zn and nitro-substituted benzenes occur inevitably and ubiquitously in aquatic environments. They have been detected simultaneously in Chinese surface water, such as Songhua River, Yellow River, and Yangtze River [25,26,27]. In this sense, the study was carried out to investigate the joint toxicity of nitro-substituted benzenes and Zn and thus to provide information for environmental pollution diagnosis and ecological risk assessment in aquatic environments. 

In the present paper, the aquatic organism, *Photobacterium phosphoreum*, was chosen as the test organism for the method is easy to perform, robust and widely used as screening method for testing toxicity of organic chemicals [28,29]. Single toxicity of nitro-substituted benzenes and Zn were determined, respectively. In order to investigate combined ratios to the joint toxic effect, mixture toxicity was determined by setting three different levels of Zn concentrations (low, medium, and high concentrations). The objectives of paper are: firstly, to evaluate the acute toxic effect of mixtures between nitro-substituted benzenes and metal Zn; secondly, to establish models and investigate the relationships between joint toxicity and calculated molecular descriptors at different Zn concentrations using the quantitative structure–activity relationships (QSARs) method, and try to analyze property or structure factors that govern the combined toxicity of nitro-substituted benzenes to *P. phosphoreum*; thirdly, to provide robust models to predict the toxicity of more nitro-substituted benzenes when they coexist with Zn.

## 2. Materials and Method

### 2.1. Chemicals

Nitro-substituted benzenes were purchased from Jilin Haotian Chemical Reagent Co. (Jilin, China) and the purity was 98% or higher. The compounds used for preparation of liquid medium were of chemically pure quality. The toxicity of Zn was evaluated using zinc (II) chloride (ZnCl_2_, purity of > 99%) which was purchased from Beijing Chemical Reagent Factory. Each stock solution of Zn or tested nitro-substituted benzenes was prepared in deionized water containing 3% NaCl so as maintain the salinity for *P. phosphoreum*, which live in a marine environment. The ultrasonic instrument (KQ3200I, made by Kunshan Ultrasonic Instrument Co., Kunshan, China) was utilized to accelerate dissolution for those organic compounds.

### 2.2. Bioassay 

Test organism, *P. phosphoreum*, appropriating for the experimental of joint effects, was served in the form of freeze-dry powder supplied by the Nanjing Institute of Soil Science, Chinese Academy of Science. The culture medium of *P. phosphoreum* was prepared according to Su et al. [20,21,22,23,24,25,26,27,28,29,30,31] and Yuan et al. [32]. The constituents of culture medium are shown in Table 1.

All the constituents were put into the volumetric flask and diluted using 100 mL deionized water. The pH of the culture medium was adjusted to 7.0 ± 0.5, then the medium was sterilized for 20 min at 121 °C. The freeze-dry powder of *P. phosphoreum* was dissolved using 0.5 mL of 3% NaCl solution, and then inoculated into 50 mL of culture medium at 21 °C.

Single toxicity of each nitro-substituted benzenes to *P. phosphoreum* or Zn was measured in the laboratory. After the preliminary experiments, each compound was set five treatment concentrations from low to high by equal interval of logarithm. Three replicates were set for each treatment concentration including the control group (solution only containing 3% NaCl in deionized water) [28,29,30,31,32,33]. For each treatment concentration, a medium solution containing diluted *P. phosphoreum* of 0.5 mL was inoculated into a tube containing 2 mL of a tested chemical solution or a 3% NaCl solution as control. After exposure of 15 min, the bioluminescence of the tested tube was detected by Microtox toxicity analyzer (DXY-2, made by the Nanjing Institute of Soil Science, Chinese Academy of Science, Nanjing, China). The inhibition rate of bioluminescence was calculated as

(1) $$\mathrm{inhibition}\;\mathrm{rate}\;(\%)\;=\;\frac{\mathrm{the}\;\mathrm{reduction}\;\mathrm{of}\;\mathrm{bioluminescence}\;\mathrm{of}\;\mathrm{each}\;\mathrm{compound}\;\mathrm{solution}}{\mathrm{the}\;\mathrm{bioluminescence}\;\mathrm{of}\;\mathrm{the}\;\mathrm{control}\;\mathrm{one}}\;\times 100\%$$

The median inhabiting concentration was expressed as IC_50_ and implies that the tested compound concentration causes a 50% inhibition on the bioluminescence of *P. phosphoreum*. The IC_50_ value (expressed in mol/L) of each tested compound and its corresponding confidence interval (CI) at 95% was calculated by method of Probit analysis [34] with the SPSS statistic package (version 19.0, SPSS Company, Chicago, IL, USA). 

To investigate the influence of combined ratios to the binary joint effects of Zn and each nitroaromatic compound towards *P. phosphoreum*, Zn concentration was set at three concentrations, 0.2, 0.5, and 0.8 ×IC_50_ of Zn (low, medium, and high levels). The mixture toxicity test was conducted by using a single concentration of Zn combined with five concentrations of the tested nitroaromatic compound which varied from low to high by equal interval of logarithm. The IC_50_ of each nitroaromatic compound at each concentration of Zn was obtained from the five concentrations of the nitroaromatic compound and percentage of inhibiting bioluminescence by method of Probit analysis. Other procedures for the joint toxicity test are the same with the individual toxicity. 

### 2.3. Calculation of Descriptors

The physico-chemical descriptors of nitro-substituted benzenes were calculated using DRAGON software (version 6.0, TALETE srl, Milano, Italy) on basis of molecular optimization [35,36]. Descriptors with constant or high pair-wise correlation were removed. A total of 361 descriptors were used for further analysis to develop QSAR models.

### 2.4. Statistical Analysis of QSAR Models

QSAR models were developed by using the stepwise linear regression method in the statistic package SPSS19.0 (SPSS Company, Chicago, IL, USA). Model quality was characterized by the number of observations (n), the square of the correlation coefficient (R^2^), the standard error of estimate (SE), Fisher’s criterion (F), and a significance level (P).

### 2.5. Evaluation Methods of Joint Toxicity

Toxic unit (TU) and additive index (AI) [37,38,39] were used in this paper to analyze and quantify the joint effects of mixtures. A TU is defined as the concentration of a particularly toxic chemical in a mixture divided by the incipient or the threshold concentration which is the exposed concentration of a chemical that causes a certain biological response end point in question (x%). This concept is mathematically expressed for the components of a mixture as formula

(2) $${\mathrm{TU}}_{i}=\frac{c_{i}}{\mathrm{EC}x_{i}}$$

where, TU_i_ is the toxic unit of chemical in an n-component mixture, c_i_ is the concentration of a chemical i that causes a certain response (x%) in that mixture, and ECx_i_ is the concentration of the chemical causing the same response when acting alone. If the sum of TU_i_ is M (M = ΣTU_i_), the additive index (AI) can be calculated according to the formulas (3) and (4)

AI = 1/M − 1.0 (M ≤ 1)(3)

AI = 1.0 − M (M > 1)(4)

The joint effects are characterized as the following: when M = 1or AI = 0, the joint effect is considered to be simple addition; when M < 1 or AI > 0, synergism; when M > 1 or AI < 0, antagonism.

## 3. Results and Discussion

### 3.1. Single Toxicity of Nitro-Substituted Benzenes and Zn

The single toxicity of 11 nitro-substituted benzenes and Zn to *P. phosphoreum* were determined and showed in Table 2. 

The result shows that the single toxicity of nitro-substituted benzenes is related to the position of substituted group. For the same substituted groups, the toxicities of *para*-substituted nitro-benzenes are more than those of *ortho*-substituted nitro-benzenes, and then *metra*-substituted nitro-benzenes. Heavy metal Zn is more toxic than 11 nitro-substituted benzenes.

### 3.2. Evaluation of Joint Toxicity of Nitro-Substituted Benzenes and Zn

**Figure 1:**
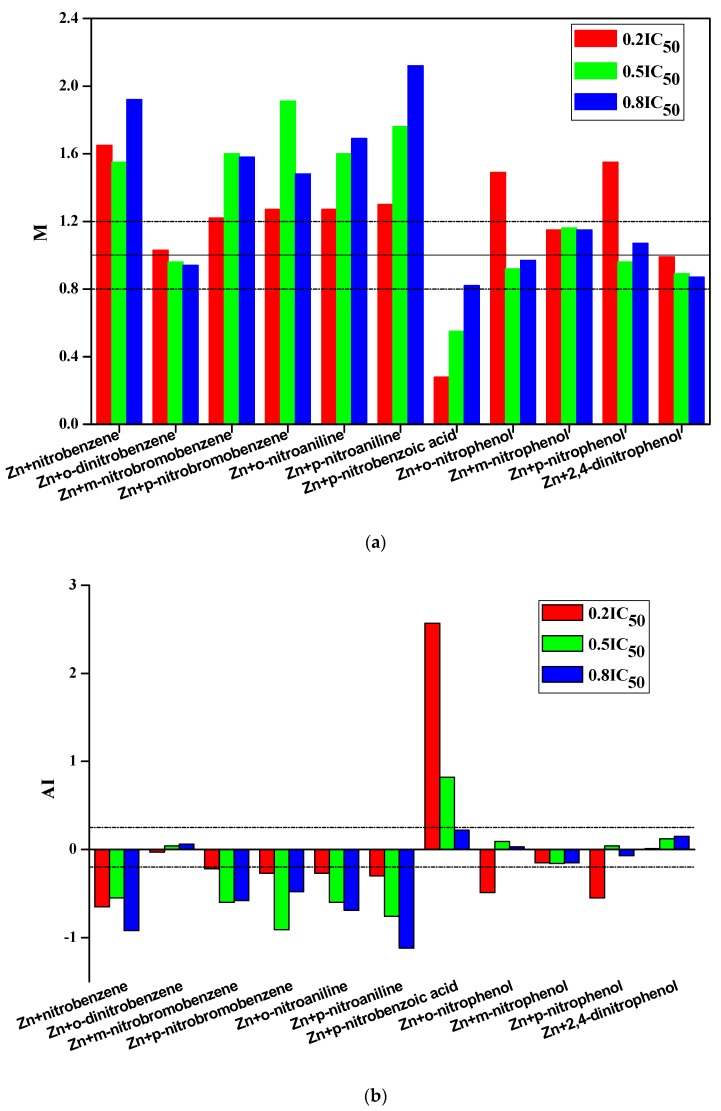
The evaluation results of joint toxicity of nitro-substituted benzenes and zinc bar by TU and AI methods ((**a**) values of M; (**b**) values of AI).

The joint toxicity of nitro-substituted benzenes when they were combined with Zn at low, medium and high concentrations was listed in Table 3. There are only two joint actions, synergism and antagonism, between nitro-substituted benzenes and Zn if we strictly obey the criteria of AI or TU (33% of the joint actions are synergism and 67% antagonism). It is widely acknowledged that within experimental error, it is an ideal situation and extremely difficult to obtain simple addition with M = 1 or AI = 0 [28,30,31,40,41]. Broderius et al. [41] defined simple addition with M values equal to 1 ± 0.2. A simple addition is characterized by 1.2 > TU (or M) > 0.8, whereas TU (or M) ≥ 1.2 represents antagonism and TU (or M) ≤ 0.8 indicates synergism in the studies of combined toxicity of antibiotics and quorum sensing inhibitors against *Escherichia coli* [28,40]. In this paper, it is referred to the methods by the above studies to define the evaluation criteria, that is, simple addition is defined as 1.2 > M > 0.8 or −0.2 < AI < 0.25. The evaluated results of the joint toxicity were shown in Figure 1.

As shown in Figure 1, at the Zn concentration of 0.2 IC_50_, the binary mixtures of Zn and 7 nitro-substituted benzenes (nitrobenzene, *m*-nitrobromobenzene, *p*-nitrobromobenzene, *o*-nitroaniline, *p*-nitroaniline, *o*-nitrophenol, *p*-nitrophenol) showed antagonism with M ranging from 1.22 to 1.65 and AI ranging from −0.65 to −0.22. The mixtures of Zn and 5 nitro-substituted benzenes (nitrobenzene, *m*-nitrobromobenzene, *p*-nitrobromobenzene, *o*-nitroaniline, *p*-nitroaniline) all showed antagonism at any Zn concentration (0.2 IC_50_, 0.5 IC_50_, or 0.8 IC_50_). Other binary mixtures are mainly simple addition. The joint toxicity of *p*-nitrobenzoic acid and Zn (at the concentration of 0.2 IC_50_ and 0.5 IC_50_) was exceptions which showed synergism. In general, the joint effects showed mainly antagonism and simple addition when Zn combined with nitro-substituted benzenes. The result is consistent with the research by Cedergreen [42] who found that synergistic interactions between chemicals are rare.

Among joint actions, synergistic interaction of chemicals in mixtures is an area of great concern to both the public [43] and regulatory authorities because some chemicals can enhance the effect of other chemicals, and they jointly exert a larger adverse effect than predicted by additive action. In this study only the mixtures of p-nitrobenzoic acid and Zn (at the concentration of 0.2 IC_50_ and 0.5 IC_50_) showed significantly synergistic interaction. The mechanism of synergism is presumably due to *p*-nitrobenzoic acid can provide an acid circumstance which can enhance the toxicity of Zn. It is also found that the average M value of -OH substituted nitorbenzene (nitrophenols) and Zn is equal to 1.10, which is lower than the one (1.62) of -NH_2_ substituted nitrobenzene and Zn. It demonstrates that the acidity will increase the joint toxicity. 

The result indicates that the joint toxicity is not only dependent on the Zn concentrations, but also on the functional groups substituted in the nitro-substituted benzenes. 

### 3.3. QSAR Analysis of the Joint Toxicity of Nitro-Substituted Benzenes and Zn

In order to identify and predict the toxicity at different combined ratios, the toxicology data of nitro-substituted benzenes combined with Zn at the concentrations of 0.2IC_50_ Zn, 0.5IC_50_ Zn, and 0.8IC_50_ Zn were analyzed with Dragon descriptors of nitro-substituted benzenes using the stepwise linear regression method in the statistic package SPSS19.0, respectively. The different linear structure–toxicity models were obtained at different combined rations of Zn and nitro-substituted benzenes.

When Zn was set at the concentration of 0.2IC_50_, the QSAR equation built from the toxicology data of nitro-substituted benzenes in mixtures and descriptors was expressed as Equation (5)

log1/IC_50_ = 20.540 − 53.948 VE2_B(p) − 0.033 TIC3 *n* = 11, R^2^ = 0.933, SE = 0.163, F = 55.554, *p* < 0.001 (5)

Two descriptors (VE2_B(p) and TIC3) were introduced to the Equation (5). The first parameter entering the equations is average coefficient of the last eigenvector from Burden matrix weighted by polarizability (VE2_B(p)) that is related to the polarizability of the molecule. It was found from Equation (4) that the toxicity of nitro-substituted benzenes in mixtures decreases with the increasing of VE2_B(p). The second is TIC3 that is total information content index (neighborhood symmetry of 3-order) which included in indices of neighborhood symmetry sub-block. 

When Zn was set at the concentration of 0.5IC_50_, the QSAR equation built from the toxicology data of nitro-substituted benzenes in mixtures and descriptors was expressed as Equation (6):

log1/IC_50_ = 3.760 + 1.100 Eig06_AEA(dm) *n* = 11, R^2^ = 0.856, SE = 0.232, F = 53.318, *p* < 0.001 (6)

Eig06_AEA(dm) standing for eigenvalue n. 6 from augmented edge adjacency matrices weighted by dipole moment is the only parameter in Equation (6). It belongs to the edge adjacency indices. Eig06_AEA(dm) is positively correlated to the joint toxicity of nitro-substituted benzenes in mixtures when Zn was set at the medium concentration. 

When Zn was set at the concentration of 0.8IC_50_, the QSAR equation built from the toxicology data of nitro-substituted benzenes in mixtures and descriptors was expressed as Equation (7):

log1/IC_50_ = 3.908 + 1.511 Eig06_AEA(dm) *n* = 11, R^2^ = 0.937, SE = 0.201, F = 133.351, *p* < 0.001 (7)

The joint toxicity of nitro-substituted benzenes is positively related to the parameter Eig06_AEA(dm) when those organic chemicals combined with Zn at high Zn concentration. Interestingly, the result showed that the same descriptor (Eig06_AEA(dm)) was chosen when nitro-substituted benzenes and Zn were combined at medium and high ratios. It implies that the toxicity of nitro-substituted benzenes correlates well with edge adjacency indices when they are combined with Zn at medium and high concentration in the present study. Whereas, it is found that other descriptors (VE2_B(p) and TIC3) are related to the toxicity of nitro-substituted benzenes in the mixture at the low concentration of Zn. It is found from Equations (5)–(7) that combined ratio is a factor which will influence the joint toxicity and should be pay more attention. The result of joint action of nitro-substituted benzenes and Zn influenced by combined ratios is in line with literature research [6,44]. Therefore, it is necessary to establish different QSAR models to predict the joint toxicity at different combined ratios. It should be noted that it is improbable to find zinc pollution exactly at 0.2, 0.5 or 0.8 times IC_50_ in real environment. The combined ratios calculated by TUs in the present study represents 3 cases theoretically, namely, nitro-substituted benzenes > Zn, nitro-substituted benzenes ≈ Zn and nitro-substituted benzenes < Zn. If nitro-substituted benzenes are more than Zn evaluated by their TUs in the real environment, descriptors of VE2_B(p) and TIC3 are more suitable to predict the toxicity; whereas, if Zn is dominant or approximately the same with nitro-substituted benzenes, descriptor Eig06_AEA(dm) is more suitable for prediction.

**Figure 2:**
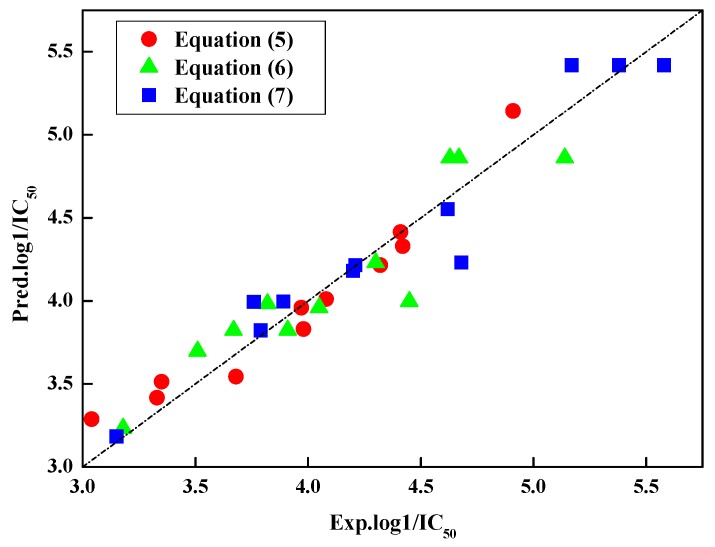
Plot of experimental and predicted values of nitro-substituted benzenes in mixtures from Equations (5)–(7).

Satisfactory models were obtained at different combined ratios of nitro-substituted benzenes and Zn with R^2^ equal to 0.933, 0.856, and 0.937, respectively. Table 4 lists the descriptors of nitro-substituted benzenes and the relative errors [28,29] from Equations (5)–(7). As is shown in Table 4, only the relative error value of *p*-nitrophenol predicted by Equation (6) is over than 10% (relative error values = 10.2%). The plot of the predicted values from Equations (5)–(7) versus the experimental values is shown in Figure 2.

Lower relative errors and good fit of predicted values and experimental values are found by Equations (5)–(7). It demonstrates that QSAR models developed in present paper are robust to predict the joint toxicity of nitro-substituted benzenes when combined with Zn at low, medium, and high concentrations.

## 4. Conclusions

The joint actions are mainly antagonism and simple addition when Zn combined with nitro-substituted benzenes. QSAR analysis shows that different descriptors could be chosen when nitro-substituted benzenes and Zn combined at low ratio. However, when nitro-substituted benzenes and Zn combined at medium and high ratios, the same descriptor was chosen in equations. At the Zn concentration of 0.2 IC_50_, the binary joint effects of Zn and nitro-substituted aromatic compounds are mainly antagonism and the joint toxicity is negatively related to the descriptors called VE2_B(p) and TIC3. At the Zn concentration of 0.5 IC_50_, the binary joint effects of Zn and nitro-substituted aromatic compounds are mainly antagonism and simple addition. At the Zn concentration of 0.8 IC_50_, the binary joint effects are antagonism (45%) and simple addition (55%). At the Zn concentration of 0.5 IC_50_ and 0.8 IC_50_, the joint toxicity are both related to the descriptor Eig06_ AEA(dm). It is found from Equations (5)–(7) that combined ratio is a factor which will influence the joint toxicity and should be pay more attention. The descriptors used are interpretable and contribute to express the joint interaction. QSAR models (Equations (5)–(7)) developed in present paper are simple, transparent and robust to predict the joint toxicity of nitro-substituted benzenes when combined with Zn at low, medium, and high concentrations.

## Figures and Tables

**Table 1:** Constituents of culture medium for *P. phosphoreum*.

No.	Compounds	Contents	No.	Compounds	Contents
1	Barm lixiviating extract	0.5 g	4	Na_2_HPO_4_	0.5 g
2	Peptone	0.5 g	5	KH_2_PO_4_	0.1 g
3	Glycerol	0.3 g	6	NaCl	3.0 g

**Table 2:** Single toxicity and corresponding confidence interval at 95% of nitro-substituted benzenes and Zn to *P. phosphoreum*.

No.	Compounds	CAS *	Experimental Single Toxicity and Corresponding Confidence Interval at 95% (log1/IC_50_)/(mol L^−1^)
1	nitrobenzene	98-95-3	3.20 (3.14–3.25)
2	*o*-dinitrobenzene	528-29-0	4.33 (4.25–4.41)
3	*m*-nitrobromobenzene	585-79-5	4.09 (4.02–4.16)
4	*p*-nitrobromobenzene	586-78-7	4.45 (4.40–4.48)
5	*o*-nitroaniline	88-74-7	3.71 (3.64–3.78)
6	*p*-nitroaniline	100-01-6	4.01 (3.98–4.04)
7	*p*-nitrobenzoic acid	62-23-7	3.81 (3.69–3.89)
8	*o*-nitrophenol	88-75-5	3.44 (3.37–3.52)
9	*m*-nitrophenol	554-84-7	3.33 (3.30–3.36)
10	*p*-nitrophenol	100-02-7	4.11 (3.99–4.23)
11	2,4-dinitrophenol	51-28-5	4.22 (4.17–4.28)
12	Zn (ZnCl_2_)	7646-85-7	5.13 (5.06–5.21)

* CAS represents the number of a chemical by Chemical Abstracts Service.

**Table 3:** Toxicity of nitro-substitutions and Zn.

Mixture	Zn (IC_50_)	Toxicity of Nitro-Substituted Benzenes in Mixtures and CI at 95% (log1/IC_50_)/(mol L^−1^)
Zn + nitrobenzene	0.2 0.5 0.8	3.04 (2.98–3.08) 3.18 (3.13–3.24) 3.15 (3.08–3.23)
Zn + *o*-dinitrobenzene	0.2 0.5 0.8	4.41 (4.34–4.48) 4.67 (4.58–4.76) 5.17 (5.07–5.31)
Zn + *m*-nitrobromobenzene	0.2 0.5 0.8	4.08 (4.01–4.14) 4.05 (3.96–4.13) 4.20 (4.11–4.30)
Zn + *p*- nitrobromobenzene	0.2 0.5 0.8	4.42 (4.38–4.45) 4.30 (4.25–5.36) 4.62 (4.43–4.83)
Zn + *o*-nitroaniline	0.2 0.5 0.8	3.68 (3.61–3.74) 3.67 (3.62–3.71) 3.76 (3.69–3.86)
Zn + *p*-nitroaniline	0.2 0.5 0.8	3.97 (3.89–4.04) 3.91 (3.87–3.93) 3.89 (3.87–3.92)
Zn + *p*-nitrobenzoic acid	0.2 0.5 0.8	4.91 (4.80–4.99) 5.14 (5.06–5.27) 5.58 (5.46–5.73)
Zn + *o*-nitrophenol	0.2 0.5 0.8	3.33 (3.21–3.45) 3.82 (3.64–3.96) 4.21 (4.02–4.42)
Zn + *m*-nitrophenol	0.2 0.5 0.8	3.35 (3.26–3.47) 3.51 (3.41–3.62) 3.79 (3.68–3.95)
Zn + *p*-nitrophenol	0.2 0.5 0.8	3.98 (4.05–4.20) 4.45 (4.33–4.51) 4.68 (4.42–4.87)
Zn + 2,4-dinitrophenol	0.2 0.5 0.8	4.32 (4.19–4.47) 4.63 (4.52–4.79) 5.38 (5.16–5.54)

**Table 4:** Descriptors of nitro-substituted benzenes and the relative errors from QSAR models.

Compounds	VE2_B(p)	TIC3	Eig06_AEA(dm)	Relative Error Values		
				Er.(1) ^a^	Er.(2) ^a^	Er.(3) ^a^
2,4-dinitrophenol	0.265	61.487	1.000	−0.024	0.050	0.007
*p*-nitrobenzoic acid	0.249	59.487	1.000	0.048	−0.054	−0.029
*o*-nitroaniline	0.282	54.000	0.057	−0.037	0.042	0.062
*o*-nitrophenol	0.284	54.603	0.203	0.026	0.042	0.001
nitrobenzene	0.295	40.548	−0.479	0.081	0.017	0.011
*p*-nitroaniline	0.278	48.000	0.058	−0.003	−0.022	0.027
*p*-nitrophenol	0.280	48.603	0.214	−0.038	−0.102	−0.096
*o*-dinitrobenzene	0.272	44.000	1.000	0.001	0.041	0.048
*m*-nitrophenol	0.281	56.603	−0.057	0.049	0.053	0.008
*m*-nitrobromobenzene	0.275	51.303	0.181	−0.017	−0.022	−0.004
*p*- nitrobromobenzene	0.274	43.303	0.427	−0.021	−0.016	−0.014

^a^ Er. (1), Er. (2), and Er. (3) are relative errors from Equations (5)–(7) which are calculated as following: Er = (V_pred._-V_exp._)/ V_exp._ (V_pred._ and V_exp._ are the experimental and predicted values, respectively).
